# Emerging roles of health information professionals for library and information science curriculum development: a scoping review

**DOI:** 10.5195/jmla.2018.354

**Published:** 2018-10-01

**Authors:** Jinxuan Ma, Lynne Stahl, Erica Knotts

**Affiliations:** School of Library and Information Management, Emporia State University, Campus Box 4025, 1 Kellogg Circle, Emporia, KS 66801; Downtown Campus Library, West Virginia University, P.O. Box 6069, 1549 University Avenue, Morgantown, WV 25606; Communication Instructor, Southern Oregon University, 1250 Siskiyou Boulevard, Ashland, OR 97504

## Abstract

**Objective:**

This scoping review identified the emerging and evolving roles of health information professionals (HIPs) in a range of tasks and settings, as they adapt to varied user needs, while keeping up with changing medical landscapes to provide evidence-based information support in grand rounds and scholarly research. The review aims to inform library school students about expected entry-level job qualifications and faculty about adaptable changes to specialized HIP curricula.

**Methods:**

The authors examined 268 peer-reviewed journal articles that concentrated on evolving HIP roles, professional settings, and contexts by retrieving results from several multidisciplinary databases.

**Results:**

HIPs, who generally serve as “embedded librarians,” are taking on more active roles as collaborators, research experts, and liaisons, replacing more passive and exclusive roles as information providers and outreach agents or research assistants. These evolving roles in the reviewed literature were broken into nine categories in approximate order of prominence.

**Conclusions:**

A new model linking these evolving roles to the Medical Library Association (MLA) fundamental professional competencies was developed to provide an operational examination and research-based evidence for adapting HIP continuing education curriculum learning outcomes, course content and delivery, and student career pathways for existing graduate HIP specialization courses in library programs. The model indicates each role’s connection to the MLA professional competencies, based on MLA’s detailed description of each competency. A better understanding of HIP demands and expectations will enhance the capacity of library programs to prepare students in HIP specializations.

## BACKGROUND

Health information professionals (HIPs), as a group of library specialists, often include “information professionals, librarians, or informaticists who have special knowledge in quality health information resources” [1]. They serve educators, students, health care providers, researchers, and the public at a time when information needs for instruction, clinical practice, research, and personal health management vary broadly. Health information services provision has become a rapidly evolving, specialized library professional field, and with this evolution comes the imperative to focus on creating and updating the cross-disciplinary curricula of professional education [2, 3].

However, it is arguable that current master’s of library science (MLS) and master’s of library and information science (MLIS) programs in North America offer insufficient academic preparation for students who wish to work in health information fields [2]. In particular, existing outdated curricula and nonspecific learning outcomes often neglect current emerging demands and challenges that health sciences libraries face [4, 5]. Further, library and information science (LIS) educators might fail to equip those students with sufficient understanding of evidence-based medicine (EBM) practice and updated HIP activities through their library science course learning [2, 3]. As a result, the disconnect between the general knowledgebase of librarianship and the specialized competency requirements for HIPs compounds the problem of inadequate guidance and instructional support for library students who are pursuing a career as an HIP and later transitioning to continuing professional development [6].

In 2017, the Medical Library Association (MLA) issued a revised set of professional competencies for HIPs, *Competencies for Lifelong Learning and Professional Success,* which includes (1) information services, (2) information management, (3) instruction and instructional design, (4) leadership and management, (5) evidence-based practice and research, and (6) health information professionalism [7]. These competencies generally emphasize community outreach, user-centered learning, current trends and climate, application of evidence-based practice principles to librarianship, and technology-enhanced learning. Specifically, the competencies address not only traditional aspects of librarianship such as library management, information provision, and user instruction, but also emerging roles and skills including open access publishing, digital preservation and organization, social media applications, and distance education.

To contextualize those professional competencies and connect them to practical health information practice, this scoping review sought to identify the evolving core HIP competencies and associated professional settings, which are fundamental to and theoretically attainable through MLS and MLIS curriculum development. It extended to peer-reviewed LIS journals as well as specialized health care journals. Two research questions were asked: How are the professional roles of HIPs and their work settings evolving based on research published since 2000? How can those identified roles be mapped onto MLA’s newly revised core competencies?

## METHODS

Journal coverage spanned fields including LIS, HIPs, health information technology, biomedicine and health sciences, clinical practice, health sciences education, and research. Frequently cited journals included but were not limited to *Health Information Library Journal,* the *Journal of Electronic Resources in Medical Libraries,* the *Journal of Hospital Librarianship,* the *Journal of the Medical Library Association, Medical Reference Services Quarterly,* and *Reference Services Review.* Databases searched included Library Information Science & Technology Abstracts (LISTA), Wilson, ScienceDirect, PubMed, and EBSCO. Search queries were tailored for different databases according to their respective controlled vocabularies:

- [“health information professional” OR “medical librarian*” AND role] for LISTA, Wilson, and ScienceDirect
- [“health information professional” OR “medical librarian*” AND “roles and responsibilities”] for ScienceDirect and EBSCO
- [“health sciences librarian*” AND role] for LISTA, Wilson, ScienceDirect, and EBSCO
- [(“libraries, medical” [MeSH] AND “librarians” [MeSH]) AND “professional role” [MeSH]] for PubMed

The inclusion criteria consisted of both experiential narratives and research articles, peer-reviewed journals, English language, North American (US/Canada), and publication dates between 2000 and 2018. With these filters, the search resulted in 750 articles. An examination using Endnote and Ulrich Serials Analysis System, supplemented by manual inspection, served to remove the duplicates and articles that did not fit the inclusion criteria (e.g., editorials and articles that focused on public libraries, which do not typically employ health information–specific librarians). The 750 articles were further scrutinized by researchers for relevance and uniqueness, with a final selection of 268 articles, managed by the citation management software Endnote, that concentrated on emerging HIP roles, professional settings, and contexts.

## RESULTS

In approximate order of frequency in or primacy of reviewed articles (n=268), the foremost roles emerged as 9 distinct categories: (1) clinical and medical information provision (n=110); (2) instruction, reference, and medical education (n=76); (3) informatics collaboration (n=74); (4) library management (n=68); (5) liaison, outreach, and inclusion (n=64); (6) research and scholarly publishing (n=64); (7) patient support and advocacy (n=62); (8) web presence and scholarly communication (n=41); and (9) data management (n=32).

The term “embedded librarians” emerged as a standout theme. While the definition varied across studies, overarching themes indicated that HIPs are taking on more active roles and functioning as partners, collaborators, research experts, and liaisons rather than more passive and exclusive roles as information providers, information outreach agents, or research assistants [8–11]. Such a generic professional role for HIPs, with its broad-ranging and loosely defined job responsibilities, makes it difficult to reach consensus when connecting embedded librarianship to required professional core competencies and fulfilling the research purposes of this study. Therefore, the duties of embedded librarians were broken down into specific roles based on their actual job responsibilities and work settings in the data analysis.

**Figure 1:**
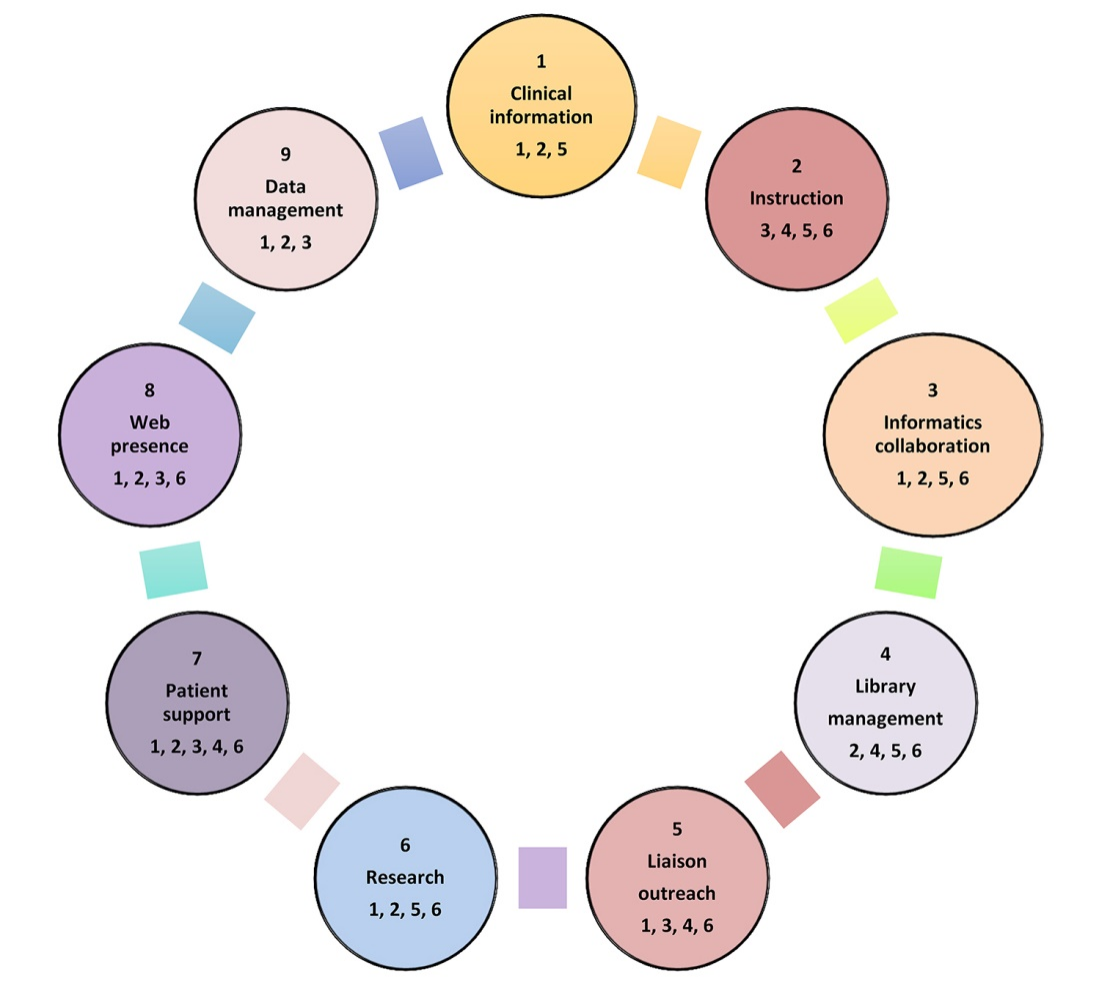
Mapping the health information professionals (HIP) roles to Medical Library Association (MLA) professional competencies For each circle, the number located above the role indicates the ranking according to the literature review. The numbers below the role indicate the MLA competencies to which the role corresponds, enumerated according to MLA [7].

The nine categories of professional roles that emerged from this review are shown in Figure 1. These professional roles or settings are ranked approximately based on their frequency and emphasis in the literature. The identified core duties of each type of professional role constitute the most common types of professional duties or responsibilities as they appear in the literature. Each is detailed in the following sections.

### 1. Clinical and medical information provision

Core duties: EBM, rounds/morning report, search support, real-time information searches, nurse support, institutional decision making

While not entirely new, clinical medical librarian roles have evolved considerably in recent years. The dual responsibilities of clinical medical librarianship require librarians to attend morning reports or rounds in which they often conduct real-time searches to support EBM practice or perform later searches based on information provided at the meeting [12, 13]. Clinical medical librarians must be equipped not only to handle specialized research inquiries based on the focus of the clinical institution or department they serve, but also to be embedded “with clinicians in processes of patient care” [14]. Although this role is still developing, it draws on librarians’ information retrieval skills to find accurate answers and evidence-based information sources for medical questions in real time or soon after, responding to the frequently pressing nature of medical information needs—whether on behalf of attending physicians, case managers, residents, interns, pharmacists, or students [14]. Moreover, the ubiquity of mobile technology has allowed clinical medical librarians to travel physically with caregiving teams to provide information in real time using networked devices [9].

As clinical medical librarians are often required to participate in medical rounds, conduct research, attend departmental events and seminars, and contribute to curriculum development for medical residency programs, their recognition as part of the patient clinical care team and as experts on health care or research teams is critical [14–16]. Clinical medical librarians should be open to new opportunities in their organizations and the roles in which they serve should be visible. They must perceive themselves as connectors in the health world to bridge the gap between medical literature and patient care [17]. Working on clinical care teams, they support patient care and attend rounds to gather information that both doctors and patients need. To aid in understanding and translating this specialized information, clinical medical librarians commonly benefit from academic qualifications or equivalent practice in the health field in addition to a traditional MLS or MLIS degree [15].

### 2. Instruction, reference, and medical education

Core duties: information literacy, continuing medical education, group and individual instruction, in-person and virtual or remote reference services, curriculum development, subject guides

Although librarians have been called on as instructors for years, their pedagogical roles have evolved considerably. Instruction has mostly moved “outside the library and becom[e] more embedded in the user’s world” [18]. With the growth of online curricula and satellite medical education programs, HIPs are expected to be able to present and maintain user engagement in active online and in-person contexts [19, 20]. Library instruction has evolved beyond just providing information to students, particularly in health sciences fields, and now entails educating users about finding credible information on their own [21]. Students receive information literacy instruction through their problem-based classroom learning when instructors allow librarians full access to the courses and present librarians to the students to better integrate them into the learning environment [22]. In this regard, instruction has become more about teaching self-sufficiency than serving as a gatekeeper of information. Librarians use a variety of pedagogical methods to deliver instruction in evidence-based practice, and further research is needed to determine the most effective methods [23].

The partnerships developed between medical instructors and librarians entail librarians not only equipping medical students with information literacy and research skills to promote lifelong learning, but also contributing to curricular instructional design and learning theory development. Specifically, health sciences librarians are often expected to attend curriculum committee meetings and reach out to students both within and outside the classroom [24], and librarians can play critical roles in developing course competencies and sharing them with other instructors [25]. Similarly, biomedical librarians are highly involved in the education process by helping to develop student skills in seeking and using quality biomedical information resources [26].

The deployment and assessment of EBM education and continuing medical education (CME) are also recognized as new roles for librarians when they serve on curriculum planning committees to identify core competencies and address knowledge gaps in professional practice [18, 27, 28]. Clinical librarians can also review periodicals to stay abreast of new techniques and technologies and alert CME staff accordingly by compiling subject guides or lists of research grant opportunities.

While many librarians are called on as partners in the classroom, either in teaching capacities or as research collaborators, many librarians receive no education or training in how to fulfill this role effectively [29]. Current MLS and MLIS programs offer few courses in instruction, and librarians are often only thrown into this role once they have entered the profession. Both the challenges that library instructors face and the importance of subject-specific training and expertise for librarians serving in these roles are recognized in the literature [29, 30]. For these reasons, many librarians pursue additional formal education to learn how to teach more effectively.

### 3. Informatics collaboration

Core duties: electronic medical records, health informatics, biomedical informatics, clinical informatics, consumer health informatics information retrieval, knowledge management

The interactions and collaboration between health sciences librarians and health informaticians regarding their trained skill sets and professional missions in health information knowledge management and dissemination are described as a “symbiotic” relationship [31]. One emerging role of health sciences librarians that is frequently addressed in the health informatics literature is their integration of evidence-based clinical resources into electronic medical records (EMRs) for both physicians and patients [32–35]. Librarians also play a crucial part in meeting other EMR needs, as EMRs must be explained and organized in a way that makes sense to staff and patients alike. Librarians’ participation lies primarily in implementing systems, creating links to the medical literature, and using EMRs to provide patient health information. For health providers, clinical decision support systems enable them to effectively support their evidence-based practice with the involvement of health sciences librarians who manage and retrieve data and information from patient EMRs [32, 36]. Those librarians may also be asked to devise “research evaluation support services” to support evidence-based practices and increasing demand to assess research output and impact [37].

For patients or health care consumers, the most important role of health sciences librarians is to facilitate access to contextualized and reliable consumer health information resources through various consumer health informatics tools. Examples of those tools include health promotion and educational materials embedded in personal health records or patient portals [38, 39]. Therefore, health sciences librarians need to serve on health informatics committees and teams to make this information accessible and intelligible to educate and assist users who are unfamiliar with the software through training and instruction [40–42].

Health sciences librarians are also called on to support the evolving needs of health informatics research, including providing information-based services for biomedical informatics and promoting cross-field collaboration in areas of knowledge management, training, and sharing of both technological and informational resources [43, 44]. HIP instruction constitutes another significant participatory role in different educational settings, such as user training for clinical informatics implementation and classroom teaching about health or medical informatics [6, 45].

### 4. Library management

Core duties: electronic resources, archives, collection development or management, technical services, programming, facility management and planning, outreach, publicity, negotiation

An observed decrease in library staffing in recent years combined with significant increases in the roles those staff are expected to play—which include providing commercial online services in addition to collection access and database or information resource training—means that librarians must be versatile and prepared for significant variation in day-to-day tasks [46]. Beyond their user-centered duties, HIPs often must assume managerial roles in their institutions. They may be asked to take measures to influence uses and perceptions of physical library buildings, working to establish these spaces as “cultural hubs” that offer activities and programs to engage broad audiences, such as conferences and symposia [47].

Similarly, they must “remain agile” and adapt to changing physical library spaces that can be used for a diverse range of purposes including collaboration, instruction, laboratory work, and research [48]. HIPs may be asked to contribute to facility planning and policy as well as to assess technological needs, signage, and architectural design [48]. For some, vendor negotiation “has become a basic skill of library acquisitions,” as libraries have in many ways become businesses with “expanding costs and shrinking budgets” [49]. As such, budding librarians on an HIP trajectory would benefit from MLS and MLIS curricula designed to develop their managerial, business negotiation, and budgeting competencies.

The research identified in this review has found disaster management to be a collaborative role in which organizations rely on librarians’ specific skills and training as a point of contact during and before an emergency. This expectation has led to HIP involvement in various contexts: assuming additional roles on emergency management teams, designing emergency management plans, and serving as a point person with connections to all other departments [17, 50–52]. Indeed, since librarians often serve as organizers and collaborators in multiple departments in hospitals, it follows logically that they would be primary contacts in the event of a disaster [52]. The role of a disaster management librarian includes providing outreach, preparedness plans, information guidelines, and service to committees and other first responders [50]. Organizations may overlook or forget the ways in which librarians can contribute to disaster planning, so it is important that librarians serve on committees and participate in plan creation, working not only to preserve relevant records, but also to serve as “information disseminators” during a disaster [50].

Finally, archives management positions and responsibilities have proliferated for many librarians in health sciences contexts since 2003, with 80% of HIP survey respondents in MLA’s Health Association and Corporate Libraries Section in 2011 reporting being responsible for archives at their institutions, compared to only 44% in a 2003 survey [53]. Respondents to Dunikowski et al.’s study, too, noted that their jobs entailed related activities such as managing web content, digitizing texts, and developing thesauri [53].

### 5. Liaison, outreach, and inclusion

Core duties: point-of-need information, instruction, continuing education, rounds or morning report institutional review, public health, cultural competency, equity and inclusion, information ethics

Increasingly, user-centered health information provision and services focusing on the unmet needs of researchers, educators, and health care providers through HIP outreach endeavors as liaisons or partners exist in all fields of biomedical research, health-related education, and clinical practice. For instance, when it comes to understanding the quality and assessment of information that patients need in clinical settings, clinicians who lack the necessary research skills and expertise may rely on librarians to assist in disseminating information and guiding individuals in locating and understanding various resources either within or outside of the organization [54]. To best provide professional health information and act as effective liaisons, librarians must remain apprised of evidence-based subject knowledge, current medical trends, and health care policy, which can include outreach and partnerships to meet the needs of underserved, marginalized, or other groups with specialized needs [55–59]. A survey by Mi and Zhang, for example, indicated that 89.1% and 93.1% of HIP respondents acknowledged the importance of culturally competent library services and health sciences librarians, respectively, to ensure that library services and information were inclusive of and accessible to user groups across racial, linguistic, sexual, and other demographics [57].

As part of the clinical care team, librarians still fulfill traditional librarian duties (including literature reviews) but largely concentrate their efforts on supporting the demands of health professionals in a liaison capacity [60–62]. With the broad range of information requests that they receive and limited resources that are available to both staff and users, librarians who do not have adequate training can feel anxiety, so preparation in MLS and MLIS curricula and beyond is crucial [61].

In academic and research settings, moreover, librarians may be called on to perform outreach, identifying and educating users on matters related to health information access, retrieval, and evaluation as well as providing customized services based on particular users’ needs and competencies [63]. These librarians can bring this inclusive, equitable mindset to important roles on institutional review boards, applying their research skills, commitment to professional ethical conduct, and neutral stance to minimize participant risk and decrease conflicts of interest in approval processes for career-shaping studies [64].

### 6. Research and scholarly publishing

Core duties: EBM practice, biomedical expertise, expert searching, reference, scholarly communications, writing skills, systematic review, citation management, copyright and intellectual property

The increasing prevalence of EBM has created a need for current, accessible health sciences research and information, leading many biomedical researchers to conduct systematic reviews—a task for which they often seek assistance from their institutions’ librarians [65–67]. This shift toward EBM has led to the development of new information service models, and the assistance that librarians provide can include information retrieval, literature review, and instruction as well as data collection and citation management [67]. A component of evidence-based librarianship, case studies have been published with increasing frequency in recent years in the *Journal of the Medical Library Association* and are popular among readers [68].

A systematic review librarian can assess and evaluate research in an organized way to meet specific criteria [18]. While this role is not new for librarians, it has become more prominent and more collaborative, as many librarians are called to serve on research teams and systematic review groups [69, 70]. Exceeding the role of an expert searcher, research and systematic review librarians often serve as liaisons to systematic research teams and assist local staff and physicians with specialized research and projects [65].

HIPs may be called upon in this capacity because, in addition to their dedication to providing information needed for informed decision making, they possess experience and skills in highly specific areas that other staff often lack. This role requires a great deal of collaboration as well as the ability to design and execute robust search processes. Systematic reviewers must be able to explore a variety of resources to find the most credible and relevant research in fields that demand a level of subject area knowledge that is deeper and further reaching than that typically encountered by other academic librarians [70, 71]. In such roles, they endeavor to bridge the gap between information and those who need it, and doing so requires a significant amount of training and experience.

Librarians also assist with miscellaneous other research-related roles, contributing to grant writing and data analysis as well as scholarly communications and translational research or the application of biomedical research to health care practice [17, 18, 72]. These key supporting roles fortify organizational efforts to secure funding and purvey academic and biomedical research to the external world. Dedicated writing time, such as the writing retreat organized by the South Central Chapter of the Medical Library Association, can have a positive impact on health sciences librarians who are pursuing research or other writing-related activities [73].

### 7. Patient support and advocacy

Core duties: reference; expert searching; knowledge brokerage; information literacy; health care policy support; policy development; cultural competency; political climate; outreach; lesbian, gay, bisexual, trans, queer (LGBTQ) competency

Because consumer health librarians can not only locate a variety of information resources, but also deliver them in a user-oriented way, they can serve the information needs of patients, their families, and the general public [74]. They also provide patient support in the form of outreach and advocacy, which require nuanced understandings of the sociopolitical and cultural climates outside of their individual institutions.

For example, two solo New England librarians reported serving as both expert searchers and knowledge brokers for patient information needs related to Medicaid policy [75]. Librarians in similar positions contributed to outreach programs for Spanish-speaking populations and facilitated access to grey literature. Further, librarians working in the University of Texas hospital system created free or low-cost informational materials for a “multi-cultural, generally low-literacy patient population”; these English-Spanish materials have since been purchased and incorporated by other libraries nationwide [76]. Librarians in such roles can collaborate closely with health care providers to clarify language and minimize patient confusion.

In public libraries, too, librarians can work to uncover specialized health information and help users to find credible information on the Internet, though they must remain within the boundaries of their job, that is, providing information but not medical advice [61]. Their role entails assisting families, patients, and the general public to locate credible health information to enable these individuals to use the information to discuss issues with medical professionals or seek out treatment [54]. HIPs in this type of position can also contribute to collection development to provide the best health information resources possible for each user and to serve as expert searchers [65, 77].

More recently, researchers discussing the growing need for culturally competent HIP librarianship cite the growing need for health information materials and services to support LGBTQ patrons or patients and their particular information needs, which necessitate that HIPs develop and practice cultural competency with regard to these communities [57–59].

### 8. Web presence and scholarly communications

Core duties: metadata, electronic resources, technical support, website planning and management, online publishing

With the growing web-based library resources available for research and publishing, librarians are able to manage and promote those digital collections with a complex skill set that encompasses organization and presentation of online information. Although literature on the topic is scarce, many HIPs are called upon to perform duties related to scholarly communications [78].

For optimal user access, they often empower users to locate, critically evaluate, and use updated evidence-based resources effectively and efficiently through sophisticated web-based information services and scholarly communications. For instance, following up on surveys from 1980, 1996, and 2003, Dunikowski et al. found that in 2011, 68% of surveyed librarians were expected to be involved in website design and planning for their institutions’ websites, compared to only 50% in 2003 [53]. New responsibilities in 2011 included metadata provision and web content archiving.

Another example is librarians’ service in emerging roles such as providing mediation for and facilitation of nurses’ online journal clubs, which are designed to improve patient outcomes and contribute to CME aspects of nursing [79]. In this and other emerging roles, librarians provided technical support, serving as website administrators, monitoring discussions, and even participating when appropriate. Librarian involvement was found to be helpful and to increase users’ awareness of web-based library resources and services [53].

### 9. Data management

Core duties: EMR compatibility and interoperability, archive management, data management, copyright and intellectual property, web content management

With advances in information science and data technology, health sciences librarianship now entails developing and managing institutional data repositories in health care organizations. Working as a data manager often requires the capacity to identify, share, manage, and preserve massive quantities of information effectively as well as knowledge of intellectual property, copyright, and permissions [18, 53, 80, 81]. Librarians may also create data dictionaries and data management plans to support institutional staff and infrastructure.

In health sciences settings, data managers assist researchers and doctors by curating and providing access to this information in an effective and understandable way. Data quantities have risen due not only to technological advances, but also to the implementation of EMRs, which must be managed and made accessible to the organization’s other health care professionals. Librarians may work to ensure compatibility and interoperability between EMR systems and library systems, implement links from EMRs to library records or from library records to point-of-care tools, or add patient and/or staff-facing contact information to EMRs [35].

Further, as data curation becomes increasingly pressing in health care fields, librarians play important embedded roles that necessitate the willingness to experiment and to work virtually in the face of shrinking budgets and limited facility space [82]. Constant and far-ranging shifts in the field of data management mean that professional development and continuing education are needed to prepare future librarians to fulfill this role and to maintain their abilities—along with a flexible mindset [35].

## DISCUSSION

In conducting this review, the goal of this review was to identify and describe emerging and evolving roles of librarians in HIP contexts between 2000 and 2018, as advances in technology and the increasing digitization of health-related information have brought significant changes to health care institutions of all types. As job expectations for HIPs change, MLS and MLIS programs must also adapt their curricula to ensure that students are prepared to fulfill expectations in entry-level jobs. This study’s alignment of MLA’s core competencies and literature detailing the many HIP roles provides an operational examination and research-based evidence for developing and updating curricular learning outcomes, course content and delivery, and student career pathways for existing graduate HIP specialization courses in library programs.

While the list of roles identified in this study is not exhaustive, it provides greater detail and expanded contexts that can inform ongoing and future curriculum development for health sciences librarianship. Mapping the identified roles to the MLA professional competencies (Figure 1) demonstrates the overlapping responsibilities and multifaceted skill sets required of health sciences librarians.

A lack of consensus and sometimes even confusion emerged in the results regarding the specific language and terminology used in either HIP job titles or HIP job descriptions related to these roles. For example, in health sciences library settings, “medical informatics” often appeared interchangeably with “library instruction focusing on information skills, technology, or medical knowledge management” [24, 31]. Indeed, King and Lapidus noted that informatics as a field itself encompasses the librarian-specific skills of research and information retrieval and evaluation [6]. Instruction in informatics can, thus, be said to overlap with general library instruction.

Acknowledging the complexities of HIP terminology, this study provides important clarification about the nature of these roles and the different contexts in which they arise. The information can be used to give MLS and MLIS students a clearer sense of what will be expected of them and to help LIS faculty develop and adapt HIP-specialization course content, delivery, learning outcomes, and practicum planning as appropriate to meet the field’s changing needs and roles.

### Mapping health information professional roles to Medial Library Association professional competencies

Many of the identified emerging roles had overlapping components, and all entailed multiple core competencies. Among the most prominent duties named in these roles were real-time search support, EBM, technological support, information literacy instruction, CME, point-of-need information, and collection development. Referencing MLA’s detailed descriptions of core professional competencies, we determined connections to relevant competencies for each of the emerging roles identified in this literature review. These connections are indicated with numerals corresponding to the respective competencies listed in the center of the model (Figure 1). The roles are assigned ordinal rankings according to frequency of occurrence in the literature. They are arranged in a circular shape to represent their connected and overlapping attributes and to avoid conveying a sense of hierarchy or the prioritization of one skill set over another.

Identifying the versatile, innovative, and challenging professional settings and practice will enrich the design of online course delivery content and strategies. By linking these evolving roles to the fundamental professional competencies, a new model was developed to improve LIS curricula for HIP (Figure 1). This proposed model will assist in providing a career pathway to guide potential library students as they consider pursuing the health information profession as their learning objectives and career goals.

### Limitations and future study

This study indicates the changes and adaptations necessary for HIP roles in the intellectually and technologically sophisticated context of health care, although it should be noted that the discussion here consists of interpretation and theorization based on an extensive review of existing literature. The research findings are not established fact but are the synthesis of a vast amount of information into an informed schema to assist library students pursuing HIP careers, LIS faculty, and employers at institutions with health information needs. HIPs are expected to perform a broad range of tasks in a variety of settings, adapting to the needs of users with language or subject knowledge barriers, while also keeping up with changing medical terminology and practice to provide search support in grand rounds and other types of research. These expectations point to areas that HIP specializations in MLS and MLIS curricula could work to address.

A follow-up examination via text mining and analysis of recent HIP job postings to specify what knowledgebases, subject knowledge, and specialized expertise a new HIP is expected to attain through preprofessional academic preparation would bridge the gap between the literature and empirical and anecdotal data. In the future, it would also be useful to examine the different kinds of coursework, training, internships, and other specialized HIP-related resources and opportunities available through existing MLS curricula at different institutions to identify areas that are lacking for students who are pursuing HIP careers.

A second phase of this study will entail administering and analyzing surveys to be completed by a range of HIP employers, including managers and administrators, medical librarians, and reference librarians at academic institutions; government, military, and private medical centers or clinics; nonprofit organizations; and research centers. A comparison between their responses and the findings from this literature review will help bridge the gap between scholarship and practice and facilitate alignment with MLA’s core competencies.

The surveys will also be useful to further identify inconsistencies between employer expectations for entry-level HIPs and what LIS curricula do to prepare students for such roles, as well as expose terminological discrepancies between the literature and employers themselves. Finally, the survey responses will shed light on soft skills, personal attributes, and non-MLS or non-MLIS educational backgrounds that create desirable candidates and competent professionals in health information contexts.

## SUPPLEMENTAL FILE

Appendix List of reviewed articles
