# Supplementary material for: Emerging roles of health information professionals for library and information science curriculum development: a scoping review
Source: J Med Libr Assoc. 2018 Oct 1;106(4):432–44. doi: 10.5195/jmla.2018.354 (PMC6148628; doi:10.5195/jmla.2018.354)
Supplement: Appendix [file jmla-106-432-s001.pdf]

JMLA Journal of the Medical Library Association

SC
Supplemental content to
J Med Libr Assoc. Oct;106(4):dx.doi.org/10.5195/jmla.2018.354
www.jmla.mlanet.org
© Ma, Stahl, Knotts 2018

Emerging roles of health information professionals for library and information science curriculum development: a scoping review

Jinxuan Ma, PhD, MLS, MA; Lynne Stahl, PhD, MLS, MA; Erica Knotts, MLS, MA

APPENDIX

List of reviewed articles

1. Aitken EM, Powelson SE, Reaume RD, Ghali WA. Involving clinical librarians at the point of care: results of a controlled intervention. Acad Med. 2011 Dec;86(12):1508–12.
2. Aken SN. Health informatics for medical librarians [review]. J Med Libr Assoc. 2010 Apr;98(2):184–6. DOI: http://dx.doi.org/10.3163/1536-5050.98.2.016.
3. Akers K, Amos K. Publishing case studies in health sciences librarianship [editorial]. J Med Libr Assoc. 2017 Apr;105(2):115–8. DOI: http://dx.doi.org/10.5195/jmla.2017.212.
4. Albert KM. Integrating knowledge-based resources into the electronic health record: history, current status, and role of librarians. Med Ref Serv Q. 2007 Fall;26(3):1–19.
5. Alcock L. Low level evidence suggests that librarian-led instruction in evidence based practice is effective regardless of instructional model. Evid Based Libr Inf Practice. 2017;12(2):163–5.
6. Aldrich AM, Schulte SJ. Establishing a new clinical informationist role in an academic health sciences center. Med Ref Serv Q. 2014;33(2):136–46.
7. Arguelles C. Evidence-based practice mentors: taking information literacy to the units in a teaching hospital. J Hosp Librariansh. 2011;11(1):8–22.
8. Atlas MC, Smigielski EM, Wulff JL, Coleman MT. Case studies from morning report: librarians' role in helping residents find evidence-based clinical information. Med Ref Serv Q. 2003 Fall;22(3):1–14.
9. Attwood CA, Wellik KE. Collaboration, collegiality, and cooperation. Clin J Oncol Nurs. 2012 Oct;16(5):487–90.
10. Avallone C. Integrating information resources and services into the electronic health record (EHR). J Med Libr Assoc. 2014 Oct;102(4):235. DOI: http://dx.doi.org/10.3163/1536-5050.102.4.003.
11. Babish J. Evidence-based medicine morning report: overview and role of the librarian. J Hosp Librariansh. 2003;3(4):35–45.
12. Banks DE, Shi R, Timm DF, Christopher KA, Duggar DC, Comegys M, McLarty J. Decreased hospital length of stay associated with presentation of cases at morning report with librarian support. J Med Libr Assoc. 2007 Oct;95(4):381–7. DOI: http://dx.doi.org/10.3163/1536-5050.95.4.381.
13. Banks MA. Defining the informationist: a case study from the Frederick L. Ehrman Medical Library. J Med Libr Assoc. 2006 Jan;94(1):5–7.
14. Banks MA, Cogdill KW, Selden CR, Cahn MA. Complementary competencies: public health and health sciences librarianship. J Med Libr Assoc. 2005 Jul;93(3):338–47.
15. Bardyn TP, Resnick T, Camina SK. Translational researchers' perceptions of data management practices and data curation needs: findings from a focus group in an academic health sciences library. J Web Librariansh. 2012;6(4):274–87.
16. Baron R, Houlihan N. Development of an ambulatory nursing newsletter: a mechanism to enhance communication. Oncol Nurs Forum. 2009;36(3):42–42.

17. Bartkowiak BA, Safford LA, Stratman EJ. Assessing the impact of a medical librarian on identification of valid and actionable practice gaps for a continuing medical education committee. J Contin Educ Health Prof. 2014 Summer;34(3):186–94. DOI: http://dx.doi.org/10.1002/chp.21244. (Subscription required.)
18. Bayrer R, Beattie S, Lucas E, Melberg D, Melton E. What have we done for you lately? measuring hospital libraries' contribution to care quality. J Hosp Librariansh. 2014;14(3):243–9.
19. Beales D. Exemplary CME: putting the 'library' into CME/library specialist. J Hosp Librariansh. 2002;2(3):29–38.
20. Beales DL. Areas for improvement in medical library advocacy: in our own words. J Hosp Librariansh. 2012;12(3):208–17.
21. Beales DL. Beyond horses to zebras: Sicca syndrome. J Hosp Librariansh. 2011;11(4):311–24.
22. Beales DL. The pharma in the dell: what medical librarians can do about the influence of industry on the provision of medical information. J Hosp Librariansh. 2007;7(4):1–14.
23. Blake L, Ballance D. Teaching evidence-based practice in the hospital and the library: two different groups, one course. Med Ref Serv Q. 2013;32(1):100–10.
24. Brackett A. Tips for the first-year health sciences librarian. Med Ref Serv Q. 2016 Oct–Dec;35(4):454–60.
25. Brady K, Kraft, M. Embedded & clinical librarianship: administrative support for vital new roles. J Libr Adm. 2012;52(8):716–30.
26. Brandes S. Experience and outcomes of medical librarian rounding. Med Ref Serv Q. 2007 Winter;26(4):85–92.
27. Brandes S, Wells K, Bandy M. Invite yourself to the table: librarian contributions to the electronic medical record. Med Ref Serv Q. 2013 Jul;32(3):358–64. DOI: http://dx.doi.org/10.1080/02763869.2013.807087.
28. Brown HA. Clinical medical librarian to clinical informationist. Ref Serv Rev. 2004;32(1):45–9.
29. Bullion J, Brower S. Enhancing the research and publication efforts of health sciences librarians via an academic writing retreat. J Med Libr Assoc. 2017 Oct;105(4):394–9. DOI: http://dx.doi.org/10.5195/jmla.2017.320.
30. Burke M, Carey P, Haines L, Lampson AP, Pond F. Implementing the information prescription protocol in a family medicine practice: a case study. J Med Libr Assoc. 2010 Jul;98(3):228–34. DOI: http://dx.doi.org/10.3163/1536-5050.98.3.011.
31. Butera G, Gomes AW, Kakar S. Expanding our roles: embedded in curriculum design. Med Ref Serv Q. 2014 Jul;33(3):292–301. DOI: http://dx.doi.org/10.1080/02763869.2014.925688.
32. Byrd GD. Can the profession of pharmacy serve as a model for health informationist professionals? J Med Libr Assoc. 2002 Jan;90(1):68–75.
33. Calabretta N. Consumer-driven, patient-centered health care in the age of electronic information. J Med Libr Assoc. 2002 Jan;90(1):32–7.
34. Carbo T. Challenges for libraries creating one world: information ethics and policy issues for medical librarians. J Med Libr Assoc. 2003 Jul;91(3):281–4.
35. Cecchino NJ. A systematic approach to developing an online medical library. J Electron Resour Med Libr. 2010;7(3):218–27.
36. Charbonneau DH. Strategies for data management engagement. Med Ref Serv Q. 2013 Jul;32(3):365–74. DOI: http://dx.doi.org/10.1080/02763869.2013.807089.

37. Chen KN, Sun HC, Lin WC, Lin PC. Into the future: three keys to success for medical libraries. J Hosp Librariansh. 2011;11(4):348–57.
38. Chimato MC, Werner SE. Building community relationships: how the library can make it happen. J Hosp Librariansh. 2006;6(2):15–25.
39. Christopher KA, Duggar DC. Librarians' role in Reach Out and Read. J Hosp Librariansh. 2008;8(3):255–63.
40. Clemmons NW, Clemmons SL. Five years later: medical reference in the 21st Century. Med Ref Serv Q. 2005 Spring;24(1):1–18.
41. Cleveland AD, Holmes KL, Philbrick JL. "Genomics and Translational Medicine for Information Professionals": an innovative course to educate the next generation of librarians. J Med Libr Assoc. 2012 Oct;100(4):303–5. DOI: http://dx.doi.org/10.3163/1536-5050.100.4.013.
42. Coady TR, Willard GK. Unlocking the power of electronic health information for public health workers in Kansas. J Med Libr Assoc. 2007 Jul;95(3):347–8. DOI: http://dx.doi.org/10.3163/1536-5050.95.3.347.
43. Cobus L. Integrating information literacy into the education of public health professionals: roles for librarians and the library. J Med Libr Assoc. 2008 Jan;96(1):28–33. DOI: http://dx.doi.org/10.3163/1536-5050.96.1.28.
44. Cooper ID. Is the informationist a new role? a logic model analysis. J Med Libr Assoc. 2011 Jul;99(3):189–92. DOI: http://dx.doi.org/10.3163/1536-5050.99.3.004.
45. Cooper ID, Crum JA. New activities and changing roles of health sciences librarians: a systematic review, 1990–2012. J Med Libr Assoc. 2013 Oct;101(4):268–77. DOI: http://dx.doi.org/10.3163/1536-5050.101.4.008.
46. Corbett M, Deardorff A, Kovar-Gough I. Emerging data management roles for health librarians in electronic medical records [reprinted from the 2013 Login Canada Student Paper Prize]. J Can Health Libr Assoc. 2014 Aug;32(2):55–9. DOI: http://dx.doi.org/10.5596/c14-022.
47. Cox AM, Corrall S. Evolving academic library specialties. J Am Soc Inf Sci Technol. 2013 Aug;64(8):1526–42.
48. Crossno JE, DeShay CH, Huslig MA, Mayo HG, Patridge EF. A case study: the evolution of a "facilitator model" liaison program in an academic medical library. J Med Libr Assoc. 2012 Jul;100(3):171–5. DOI: http://dx.doi.org/10.3163/1536-5050.100.3.006.
49. Crum JA, Cooper ID. Emerging roles for biomedical librarians: a survey of current practice, challenges, and changes. J Med Libr Assoc. 2013 Oct;101(4):278–86. DOI: http://dx.doi.org/10.3163/1536-5050.101.4.009.
50. Crumley ET. Exploring the roles of librarians and health care professionals involved with complementary and alternative medicine. J Med Libr Assoc. 2006 Jan;94(1):81–9.
51. Cruse P, Protzko S. Librarian contributions to clinical practice guidelines. Med Ref Serv Q. 2014 Jul;33(3):327–34. DOI: http://dx.doi.org/10.1080/02763869.2014.925710.
52. Curtis JA. Electronic health records, platforms, libraries, and evidence: report on the Association of Academic Health Sciences Libraries symposium's keynote presentation by Kenneth Mandl. J Med Libr Assoc. 2010 Jul;98(3):206–9. DOI: http://dx.doi.org/10.3163/1536-5050.98.3.005.
53. Dalrymple PW, Roderer NK. Education for health information professionals: perspectives from health informatics in the U.S. Educ Inf. 2010 Mar;28(1):45–55.
54. Davies KJ, Blake L, Palladino C, Halbert J. Designing a DREAM database: library faculty integration in medical education assessment. J Med Libr Assoc. 2014 Oct;102(4):234–5. DOI: http://dx.doi.org/10.3163/1536-5050.102.4.003.

SC
Supplemental content to
J Med Libr Assoc. Oct;106(4):dx.doi.org/10.5195/jmla.2018.354
www.jmla.mlanet.org
© Ma, Stahl, Knotts 2018

55. Deberg J, Egeland M. Hospital noise: how librarians can help. J Hosp Librariansh. 2014;14(2):120–39.
56. De Jager-Loftus D, Midyette JD, Harvey B. A community of practice: librarians in a biomedical research network. Med Ref Serv Q. 2014 Jan;33(1):60–74.
57. Dell EY, Shultz SM. Conserving digital resources: issues and future access. J Electron Resour Med Libr. 2014;11(3):124–33.
58. Detlefsen EG. Clinical research informationist. Ref Serv Rev. 2004;32(1):26–30.
59. Detlefsen EG. The education of informationists, from the perspective of a library and information sciences educator. J Med Libr Assoc. 2002 Jan;90(1):59–67.
60. Detlefsen EG. Teaching about teaching and instruction on instruction: a challenge for health sciences library education. J Med Libr Assoc. 2012 Oct;100(4):244–50. DOI: http://dx.doi.org/10.3163/1536-5050.100.4.005.
61. Devin RB. Doing without: serving allied health programs at universities without medical schools. Med Ref Serv Q. 2009 Spring;28(1):44–55.
62. Donahue AE, Featherstone RM. New roles for hospital librarians: a benchmarking survey of disaster management activities. J Med Libr Assoc. 2013 Oct;101(4):315–8. DOI: http://dx.doi.org/10.3163/1536-5050.101.4.014.
63. Dorsch JL, Perry GJ. Evidence-based medicine at the intersection of research interests between academic health sciences librarians and medical educators: a review of the literature. J Med Libr Assoc. 2012 Oct;100(4):251–7. DOI: http://dx.doi.org/10.3163/1536-5050.100.4.006.
64. Doyle JD, Harvey SA. Teaching the publishing process to researchers and other potential authors in a hospital system. J Hosp Librariansh. 2005;5(1):63–70.
65. Droese P, Peterson N. Utilization of the medical librarian in a state Medicaid program to provide information services geared to health policy and health disparities. J Med Libr Assoc. 2006 Apr;94(2):174–9.
66. Dudden RF, Protzko SL. The systematic review team: contributions of the health sciences librarian. Med Ref Serv Q. 2011 Jul;30(3):301–15.
67. Duncan S. Instructional design for librarians and information professionals. J Med Libr Assoc. 2013 Jan;101(1):77–8. DOI: http://dx.doi.org/10.3163/1536-5050.101.1.013.
68. Dunikowski LG, Embrey AC, Hawkes WG, Riedlinger JE, Taliaferro MG, Van Hine PM. The Health Association Libraries Section survey: finding clues to changing roles. J Med Libr Assoc. 2013 Oct;101(4):318–22. DOI: http://dx.doi.org/10.3163/1536-5050.101.4.015.
69. Egan L. The librarian as a member of the education department team: using Web 2.0 technologies to improve access to education materials and information. Med Ref Serv Q. 2012;31(3):330–5.
70. Egeland M. Hospital librarians: from consumer health to patient education and beyond. J Hosp Librariansh. 2015 Jan;15(1):65–76. DOI: http://dx.doi.org/10.1080/15323269.2015.982944.
71. Eldredge JD. Evidence-based librarianship: searching for the needed EBL evidence. Med Ref Serv Q. 2000 Fall;19(3):1–18.
72. Ellero NP. Crossing over: health sciences librarians contributing and collaborating on electronic medical record (EMR) implementation. J Hosp Librariansh. 2009;9(1):89–107.
73. Elliott KJ. Genomic medicine enters the clinic: new roles and challenges for hospital librarians. J Hosp Librariansh. 2012;12(4):372–7.
74. Esparza JM, Shi R, McLarty J, Comegys M, Banks DE. The effect of a clinical medical librarian on in-patient care outcomes. J Med Libr Assoc. 2013 Jul;101(3):185–91. DOI: http://dx.doi.org/10.3163/1536-5050.101.3.007.

75. Featherstone RM. The disaster information specialist: an emerging role for health librarians. J Libr Adm. 2012 Nov;52(8):731–53. DOI: http://dx.doi.org/10.1080/01930826.2012.746875.
76. Featherstone RM, Boldt RG, Torabi N, Konrad SL. Provision of pandemic disease information by health sciences librarians: a multisite comparative case series. J Med Libr Assoc. 2012 Apr;100(2):104–12. DOI: http://dx.doi.org/10.3163/1536-5050.100.2.008.
77. Federer L. The librarian as research informationist: a case study. J Med Libr Assoc. 2013 Oct;101(4):298–302. DOI: http://dx.doi.org/10.3163/1536-5050.101.4.011.
78. Fell DW, Burnham JF. Access is key: teaching students and physical therapists to access evidence, expert opinion, and patient values for evidence-based practice. J Phys Ther Educ. 2004;18(3):12–23.
79. Fell DW, Burnham JF, Dockery JM. Determining where physical therapists get information to support clinical practice decisions. Health Inf Libr J. 2013;30(1):35–48.
80. Fong BL, Wang M, White K, Tipton R. Assessing and serving the workshop needs of graduate students. J Acad Librariansh. 2016;42(5):569–80.
81. Foster J, Flanders S. Challenges in clinical nurse specialist education and practice. Online J Issues Nurs. 2014 May 31;19(2):1.
82. Foster MJ, Shurtz S, Pepper C. Evaluation of best practices in the design of online evidence-based practice instructional modules. J Med Libr Assoc. 2014 Jan;102(1):31–40. DOI: http://dx.doi.org/10.3163/1536-5050.102.1.007.
83. Fowler SA, Yaeger LH, Yu F, Doerhoff D, Schoening P, Kelly B. Electronic health record: integrating evidence-based information at the point of clinical decision making. J Med Libr Assoc. 2014 Jan;102(1):52–5. DOI: http://dx.doi.org/10.3163/1536-5050.102.1.010.
84. Freiburger G, Martin JR, Nuñez AV. An embedded librarian program: eight years on. Med Ref Serv Q. 2016 Oct–Dec;35(4):388–96.
85. Fuller SS. Enabling, empowering, inspiring: research and mentorship through the years. Bull Med Libr Assoc. 2000 Jan;88(1):1–10.
86. Garcia JL, Wells KK. Knowledge-based information to improve the quality of patient care. J Healthc Quality. 2009 Jan–Feb;31(1):30–5.
87. Gaspard CS, Prentice KA. Focused library instruction for occupational therapy, physical therapy, and respiratory care students and faculty: library instruction in allied health. Med Ref Serv Q. 2014;33(3):335–42.
88. Geer RC. Broad issues to consider for library involvement in bioinformatics. J Med Libr Assoc. 2006 Jul;94(3):286–98.
89. Geng Y, Fowler CS, Fulton S. Clinical librarian support for rapid review of clinical utility of cancer molecular biomarkers. Med Ref Serv Q. 2015;34(2):202–14.
90. Gillum S, Chiplock A. How to build successful iPad programs in health science libraries: a tale of two libraries. J Electron Res Med Libr. 2014;11(1):29–38.
91. Giuse NB, Koonce TY, Jerome RN, Cahall M, Sathe NA, Williams A. Evolution of a mature clinical informationist model. J Am Med Inform Assoc. 2005 May–Jun;12(3):249–55.
92. Giuse NB, Kusnoor SV, Koonce TY, Ryland CR, Walden RR, Naylor HM, Williams Am, Jerome RN. Strategically aligning a mandala of competencies to advance a transformative vision. J Med Libr Assoc. 2013 Oct;101(4):261–7. DOI: http://dx.doi.org/10.3163/1536-5050.101.4.007.
93. Gluck JC, Hassig RA. Raising the bar: the importance of hospital library standards in the continuing medical education accreditation process. Bull Med Libr Assoc. 2001 Jul;89(3):272–6.

94. Goben A, Raszewski R. The data life cycle applied to our own data. J Med Libr Assoc. 2015 Jan;103(1):40–4. DOI: http://dx.doi.org/10.3163/1536-5050.103.1.008.
95. Gomes A, Palena-Hall E, Abate L, Sullo E, Levett P, Wilcoxson M. Connecting to our community: extending librarians' roles through collaboration. J Hosp Librariansh. 2011;11(2):165–74.
96. Gore SA. E-science and data management resources on the web. Med Ref Serv Q. 2011;30(2):167–-77.
97. Grandage KK, Slawson DC, Shaughnessy AF. When less is more: a practical approach to searching for evidence-based answers. J Med Libr Assoc. 2002 Jul;90(3):298–304.
98. Greyson D, Surette S, Dennett L, Chatterley T. "You're just one of the group when you're embedded": report from a mixed-method investigation of the research-embedded health librarian experience. J Med Libr Assoc. 2013 Oct;101(4):287–97. DOI: http://dx.doi.org/10.3163/1536-5050.101.4.010.
99. Griffin-Sobel JP, Acee A, Sharoff L, Cobus-Kuo L, Woodstock-Wallace A, Dornbaum M. A transdisciplinary approach to faculty development in nursing education technology. Nurs Educ Perspect. 2010 Jan–Feb;31(1):41–3.
100. Gu XM. Librarians' roles in evidence-based dentistry education: a review of literature and a survey in North America. Med Ref Serv Q. 2010 Oct;29(4):331–48.
101. Guerrieri R. Librarian participation in expanding the pool of potential medical students. Med Ref Serv Q. 2012;31(1):61–72.
102. Gutzman KE, Bales ME, Belter CW, Chambers T, Chan L, Holmes KL, Lu YL, Palmer LA, Reznik-Zellen RC, Sarli CC, Suiter AM, Wheeler TR. Research evaluation support services in biomedical libraries. J Med Libr Assoc. 2018 Jan;106(1):1–14. DOI: http://dx.doi.org/10.5195/jmla.2018.205.
103. Haines M, Marshall JG. Future of academic and health libraries: personal perspectives. Health Inf Libr J. 2008;25(suppl 1):94–5.
104. Hammond PA. Immersed in patient care: mission critical decisions for hospital libraries. Against Grain. 2011;23(6):22–6.
105. Harris MR. The librarian's roles in the systematic review process: a case study. J Med Libr Assoc. 2005 Jan;93(1):81–7.
106. Harris R, Mayo A, Prince JD, Tooey MJ. Creating shared campus experiences: the library as culture club. J Med Libr Assoc. 2013 Oct;101(4):254–6. DOI: http://dx.doi.org/10.3163/1536-5050.101.4.005.
107. Harvey S. Institutional review boards: another way for hospital librarians to add value to their organization. J Hosp Librariansh. 2003 Jun;3(2):99–102.
108. Hasman L. Librarian-facilitated problem-based learning course in a school of dental medicine. Med Ref Serv Q. 2012;31(3):336–41.
109. Hauser SE, Demner-Fushman D, Jacobs JL, Hunphrey SM, Ford G, Thoma GR. Using wireless handheld computers to seek information at the point of care: an evaluation by clinicians. J Am Med Inf Assoc. 2007 Nov–Dec;14(6):807–15.
110. Hawkins BW, Morris M, Nguyen T, Siegel J, Vardell E. Advancing the conversation: next steps for lesbian, gay, bisexual, trans, and queer (LGBTQ) health sciences librarianship. J Med Libr Assoc. 2017 Oct; 105(4):316–27. DOI: http://dx.doi.org/10.5195/jmla.2017.206.
111. Helms AJ, Bradford KD, Warren NJ, Schwartz DG. Bioinformatics opportunities for health sciences librarians and information professionals. J Med Libr Assoc. 2004 Oct;92(4):489–93.
112. Hersh W. Ubiquitous but unfinished: grand challenges for information retrieval. Health Inf Libr J. 2008;25(suppl 1):90–3.

113. Hersh W. Who are the informaticians? what we know and should know. J Am Med Inf Assoc. 2006 Mar–Apr;13(2):166–70.
114. Holst R, Funk CJ. State of the art of expert searching: results of a Medical Library Association survey. J Med Libr Assoc. 2005 Jan;93(1):45–52.
115. Holst R, Funk CJ, Adams HS, Bandy M, Boss CM, Hill B, Joseph CB, Lett RK. Vital pathways for hospital librarians: present and future roles. J Med Libr Assoc. 2009 Oct;97(4):285–92. DOI: http://dx.doi.org/10.3163/1536-5050.97.4.013.
116. Homan JM. Eyes on the prize: reflections on the impact of the evolving digital ecology on the librarian as expert intermediary and knowledge coach, 1969–2009. J Med Libr Assoc. 2010 Jan;98(1):49–56. DOI: http://dx.doi.org/10.3163/1536-5050.98.1.016.
117. Homan JM, McGowan JJ. The Medical Library Association: promoting new roles for health information professionals. J Med Libr Assoc. 2002 Jan;90(1):80–5.
118. Hoy MB. Personal activity trackers and the quantified self. Med Ref Serv Q. 2016;35(1):94–100.
119. Huber JT, Shapiro II RM, Burke HJ, Palmer A. Enhancing the care navigation model: potential roles for health sciences librarians. J Med Libr Assoc. 2014 Jan;102(1):55–61. DOI: http://dx.doi.org/10.3163/1536-5050.102.1.011.
120. Huffman IR, Martin HJ, Delawska-Elliott B. Creating a library holding group: an approach to large system integration. J Med Libr Assoc. 2016 Oct;104(4):315–9. DOI: http://dx.doi.org/10.3163/1536-5050.104.4.013.
121. Hurst EJ. Educational technologies in health sciences libraries: teaching technology skills. Med Ref Serv Q. 2014;33(1):102–8.
122. Jenkins CG, Bader SA. The Association of Academic Health Sciences Libraries' collaboration with the Association of American Medical Colleges, Medical Library Association, and other organizations. J Med Libr Assoc. 2003 Apr;91(2):161–7.
123. Johnston J. The effectiveness of online instruction: a literature review. Can J Med Radiat Technol. 2007;38(2):17–21.
124. Jones DA, Shipman JP, Plaut DA, Selden CR. Characteristics of personal health records: findings of the Medical Library Association/National Library of Medicine Joint Electronic Personal Health Record Task Force. J Med Libr Assoc. 2010 Jul;98(3):243–9. DOI: http://dx.doi.org/10.3163/1536-5050.98.3.013.
125. Kaplan R, Steinberg M, Doucette J. Retention of retrospective print journals in the digital age: trends and analysis. J Med Libr Assoc. 2006 Oct;94(4):387–93, e198–e200.
126. Kean EB. Assessment and impact of a new role as an embedded librarian in nursing online journal clubs. J Med Libr Assoc. 2013 Oct;101(4):335–8. DOI: http://dx.doi.org/10.3163/1536-5050.101.4.018.
127. Kendall SK. Basic biomedical scientists: the rediscovered library users. Against Grain. 2014;26(2):34–6.
128. Kendall SK. Strategies of health sciences librarians working without a traditional health sciences library. J Hosp Librariansh. 2012;12(4):363–71.
129. Kennedy MG, Kiken L, Shipman JP. Addressing underutilization of consumer health information resource centers: a formative study. J Med Libr Assoc. 2008 Jan;96(1):42–9. DOI: http://dx.doi.org/10.3163/1536-5050.96.1.42.
130. Keselman A, Quasem S, Kelly JE, Dutcher GA. Evaluation of a health sciences internship for Latino and Native American library students. J Med Libr Assoc. 2016 Oct;104(4):328–32. DOI: http://dx.doi.org/10.3163/1536-5050.104.4.016.

131. Kies S, Shultz M. Proposed changes to the United States Medical Licensing Examination: impact on curricula and libraries. J Med Libr Assoc. 2010 Jan;98(1):12–6. DOI: http://dx.doi.org/10.3163/1536-5050.98.1.007.
132. King DN. The contribution of hospital library information services to clinical care: a study in eight hospitals. J Med Libr Assoc. 2012 Oct;100(4 suppl):H.
133. King SB, Lapidus M. Metropolis revisited: the evolving role of librarians in informatics education for the health professions. J Med Libr Assoc. 2015 Jan;103(1):14–8. DOI: http://dx.doi.org/10.3163/1536-5050.103.1.003.
134. Klein-Fedyshin M. It was the worst of times, it was the best of times: positive trends influencing hospital libraries. J Med Libr Assoc. 2010 Jul;98(3):196–9. DOI: http://dx.doi.org/10.3163/1536-5050.98.3.002.
135. Klein-Fedyshin M, Burda ML, Epstein BA, Lawrence B. Collaborating to enhance patient education and recovery. J Med Libr Assoc. 2005 Oct;93(4):440–5.
136. Klem ML, Weiss PM. Evidence-based resources and the role of librarians in developing evidence-based practice curricula. J Prof Nurs. 2005 Nov–Dec;21(6):380–7.
137. Knight T, Brice A. Librarians, surgeons, and knowledge. Surg Clin North Am. 2006 Feb;86(1):71–90, viii–ix.
138. Koonce TY, Giuse DA, Beauregard JM, Giuse NB. Toward a more informed patient: bridging health care information through an interactive communication portal. J Med Libr Assoc. 2007 Jan;95(1):77–81.
139. Kotzin S, Lyon B. Leading into the future: library operations at the National Library of Medicine. J Med Libr Assoc. 2008 Jan;96(1):1–2. DOI: http://dx.doi.org/10.3163/1536-5050.96.1.1.
140. Krieger MM, Richter RR, Austin TM. An exploratory analysis of PubMed's free full-text limit on citation retrieval for clinical questions. J Med Libr Assoc. 2008 Oct;96(4):351–5. DOI: http://dx.doi.org/10.3163/1536-5050.96.4.010.
141. Krom ZR, Batten J, Bautista C. A unique collaborative nursing evidence-based practice initiative using the Iowa model: a clinical nurse specialist, a health science librarian, and a staff nurse's success story. Clin Nurse Spec. 2010 Mar–Apr;24(2):54–9.
142. Kronenfeld M, Doyle JD. From MEDLINE gatekeeper to KBI portal: a new model for hospital libraries. J Hosp Librariansh. 2003;3(2):1–18.
143. Kwasik H, Fulda PO. Strengthening professionals: a chapter-level formative evaluation of the Medical Library Association mentoring initiative. J Med Libr Assoc. 2006 Jan;94(1):19–29.
144. Kyne A. Charting consumer health. improving users' ability to find online health resources: creating search-engine-friendly web pages. J Hosp Librariansh. 2004;4(4):65–71.
145. Landau R. Solo librarian and outreach to hospital staff using Web 2.0 technologies. Med Ref Serv Q. 2010 Jan;29(1):75–84.
146. Lapidus M, Kostka-Rokosz MD, Dvorkin-Camiel L. Librarian-lead tutorial for enhancement of pharmacy students' information-searching skills in advanced experiential rotations. Med Ref Serv Q. 2009 Oct;28(4):351–62.
147. Lappa E. Different information needs of emergency department staff: getting relevant evidence from a clinical librarian program. J Hosp Librariansh. 2004;4(3):35–52.
148. Leisey MR. Viewpoints from a social work information specialist in context: thoughts for consumer health librarians. J Consumer Health Internet. 2007;11(4):15–22.

149. Lemkau HL Jr. Constants, context, and change: the pursuit of purpose. J Med Libr Assoc. 2008 Jan;96(1):12–9. DOI: http://dx.doi.org/10.3163/1536-5050.96.1.12.
150. Lessick S. Tooling up to facilitate findability, virtual collaboration, and storytelling with data. J Med Libr Assoc. 2016 Oct;104(4):354–62. DOI: http://dx.doi.org/10.3163/1536-5050.104.4.021.
151. Levin RF. Developing the infrastructure to support EBP: it takes a library. Res Theory Nurs Pract. 2007;21(2):77–9.
152. Linton AM. Emerging roles for librarians in the medical school curriculum and the impact on professional identity. Med Ref Serv Q. 2016 Oct–Dec;35(4):414–33.
153. Lobach DF. Clinical informatics: supporting the use of evidence in practice and relevance to physical therapy education. J Phys Ther Educ. 2004 Dec;18(3):24–34.
154. Locatis C, Gaines C, Wei-Li L, Gill M, Ackerman M. Lessons learned from ten years of distance learning outreach. J Med Libr Assoc. 2015 Apr;103(2):78–82. DOI: http://dx.doi.org/10.3163/1536-5050.103.2.004.
155. Lovett DG. PDAs @ the library. PDAs: exploring the role of the medical librarian. J Electron Resour Med Libr. 2004;1(1):113–8.
156. Ludeman E, Downton K, Shipper AG, Fu Y. Developing a library systematic review service: a case study. Med Ref Serv Q. 2015;34(2):173–80.
157. Ludwig L, Starr S. Library as place: results of a delphi study. J Med Libr Assoc. 2005 Jul;93(3):315–26.
158. Lynn VA, FitzSimmons M, Robinson CK. Special report: symposium on transformational change in health sciences libraries: space, collections, and roles. J Med Libr Assoc. 2011 Jan;99(1):82–7. DOI: http://dx.doi.org/10.3163/1536-5050.99.1.014.
159. Lyon JA, Tennant MR, Messner KR, Osterbur DL. Carving a niche: establishing bioinformatics collaborations. J Med Libr Assoc. 2006 Jul;94(3):330–5.
160. MacDonald KA, Hrynchak PK, Spafford MM. Evidence-based practice instruction by faculty members and librarians in North American optometry and ophthalmology programs. J Med Libr Assoc. 2014 Jul;102(3):210–5. DOI: http://dx.doi.org/10.3163/1536-5050.102.3.013.
161. Maggio LA, Durieux N, Tannery NH. Librarians in evidence-based medicine curricula: a qualitative study of librarian roles, training, and desires for future development. Med Ref Serv Q. 2015;34(4):428–40. DOI: http://dx.doi.org/10.1080/02763869.2015.1082375.
162. Maggio LA, Posley KA. Training the trainers: teaching clinician educators to provide information literacy skills feedback. J Med Libr Assoc. 2011 Jul;99(3):258–61. DOI: http://dx.doi.org/10.3163/1536-5050.99.3.014.
163. Marshall JG, Sollenbergers J, Easterby-Gannett S, Morgan L, Klem ML, Cavanaugh SK, Oliver KB, Thompson CA, Romanosky N, Hunter S. The value of library and information services in patient care: results of a multisite study. J Med Libr Assoc. 2013 Jan;101(1):38–46. DOI: http://dx.doi.org/10.3163/1536-5050.101.1.007.
164. Martin ER. Shaping opportunities for the new health sciences librarian. J Med Libr Assoc. 2013 Oct;101(4):252–3. DOI: http://dx.doi.org/10.3163/1536-5050.101.4.004.
165. Matheson NW, Welch WH. The idea of the library in the twenty-first century. J Med Libr Assoc. 2012 Oct;100(4 suppl):I.
166. Mayer S, Smith KH, Rios G. Consumer health information services 2.0. J Consumer Health Internet. 2008;12(3):187–99.
167. Mayo HG, Pestonjee SF. Creating understandable health information. J Hosp Librariansh. 2004 Dec;4(4):113–8. DOI: http://dx.doi.org/10.1300/J186v04n04_10.

168. Mazure ES, Alpi KM. Librarian readiness for research partnerships. J Med Libr Assoc. 2015 Apr;103(2):91–5. DOI: http://dx.doi.org/10.3163/1536-5050.103.2.007.
169. McClure LW. When the librarian was the search engine: introduction to the special issue on new roles for health sciences librarians. J Med Libr Assoc. 2013 Oct;101(4):257–60. DOI: http://dx.doi.org/10.3163/1536-5050.101.4.006.
170. McMullen KD, Yeh, F. Adapting to change: a survey of evolving job descriptions in medical librarianship. J Hosp Librariansh. 2013;13(3):246–57.
171. Mears K, Bandy S. Investigating the need for scholarly communications positions in Association of Academic Health Sciences Libraries member institutions. J Med Libr Assoc. 2017 Apr;105(2):145–9. DOI: http://dx.doi.org/10.5195/jmla.2017.208.
172. Meert D, Torabi N, Costella J. Impact of librarians on reporting of the literature searching component of pediatric systematic reviews. J Med Libr Assoc. 2016 Oct;104(4):267–77. DOI: http://dx.doi.org/10.3163/1536-5050.104.4.004.
173. Meng L, Yi-Bu C, Clintworth WA. Expanding roles in a library-based bioinformatics service program: a case study. J Med Libr Assoc. 2013 Oct;101(4):303–9. DOI: http://dx.doi.org/10.3163/1536-5050.101.4.012.
174. Mi M. Leveraging research synthesis for promoting and expanding library services and educational programs. J Acad Librariansh. 2016;42(2):151–3.
175. Mi M, Zhang Y. Culturally competent library services and related factors among health sciences librarians: an exploratory study. J Med Libr Assoc. 2017 Apr;105(2):132–9. DOI: http://dx.doi.org/10.5195/jmla.2017.203.
176. Miller JM. A framework for the multiple roles of librarians in problem-based learning. Med Ref Serv Q. 2001 Fall;20(3):23–30.
177. Miller JM. Issues surrounding the administration of a credit course for medical students: survey of US academic health sciences librarians. J Med Libr Assoc. 2004 Jul;92(3):354–63.
178. Morgen EB. Implementing PDA technology in a medical library: experiences in a hospital library and an academic medical center library. Med Ref Serv Q. 2003 Spring;22(1):11–9.
179. Morley SK, Buchanan HS. Clinical medical librarians: extending library resources to the clinical setting. J Hosp Librariansh. 2001;1(2):15–30.
180. Morris M, Hawkins B. Towards a new specialization in health librarianship: LGBTQ health. J Can Health Libr Assoc. 2016 Apr;37(1):20–3. DOI: http://dx.doi.org/10.5596/c16-007.
181. Murphy J. Globalization: implications for health information professionals. Health Inf Libr J. 2008;25(1):62–8.
182. Murphy J, Adams A. Exploring the benefits of user education: a review of three case studies. Health Inf Libr J. 2005;22(suppl 1):45–58.
183. Murphy SA, Boden C. Benchmarking participation of Canadian university health sciences librarians in systematic reviews. J Med Libr Assoc. 2015 Apr;103(2):73–8. DOI: http://dx.doi.org/10.3163/1536-5050.103.2.003.
184. Myers BA, Rodriguez B. How do early career health sciences information professionals gain competencies? J Med Libr Assoc. 2016 Jul;104(3):215–20. DOI: http://dx.doi.org/10.3163/1536-5050.104.3.006.
185. Nix AT, Huber JT, Shapiro RM II, Pfeifle A. Examining care navigation: librarian participation in a team-based approach? J Med Libr Assoc. 2016 Apr;104(2):131–7. DOI: http://dx.doi.org/10.3163/1536-5050.104.2.007.

186. Obrig KS, Thompson JL. Changing titles, changing duties: restructuring the role of the serials librarian for an electronic future. J Electron Resour Med Libr. 2005;2(2):59–68. DOI: https://dx.doi.org/10.1300/J383v02n02_06.
187. Odell E, Barta K. Teaching evidence-based practice: the bachelor of science in nursing essentials at work at the bedside. J Prof Nurs. 2011 Nov–Dec;27(6):370–7.
188. Oduwole AA, Olatundun OO. Electronic technology tools for knowledge management by health information professionals. J Hosp Librariansh. 2010;10(3):305–14.
189. Oliver KB, Dalrymple P, Lehmann HP, McClellan DA, Robinson KA, Twose C. Bringing evidence to practice: a team approach to teaching skills required for an informationist role in evidence-based clinical and public health practice. J Med Libr Assoc. 2008 Jan;96(1):50–7. DOI: http://dx.doi.org/10.3163/1536-5050.96.1.50.
190. Oliver KB, Roderer NK. Working towards the informationist. Health Inf J. 2006;12(1):41–8.
191. Orick JT. The business of negotiating for hospital librarians. Med Ref Serv Q. 2004 Fall;23(3):61–9.
192. Pappas C. Hospital librarians' perceptions related to evidence-based health care. J Med Libr Assoc. 2008 Jul;96(3):235–8. DOI: http://dx.doi.org/10.3163/1536-5050.96.3.011.
193. Paradise A. Why the Joint Commission on Accreditation of Healthcare Organizations should add new regulations regarding libraries. J Med Libr Assoc. 2004 Apr;92(2):166–8.
194. Perley CM, Gentry CA, Fleming AS, Sen KM. Conducting a user-centered information needs assessment: the Via Christi Libraries' experience. J Med Libr Assoc. 2007 Apr;95(2):173–81, e54–e55.
195. Perry G, Weldon S. Consumer health informatics research: implications for consumers, health information professionals, and researchers. J Consumer Health Internet. 2005;9(2):1–10.
196. Perry GJ. Viva la evolution! specialized adaptation and the Medical Library Association's ongoing commitment to the teaching and learning roles of health sciences librarians [editorial]. J Med Libr Assoc. 2013 Jan;101(1):1–3. DOI: http://dx.doi.org/10.3163/1536-5050.101.1.001.
197. Perry GJ, Kronenfeld MR. Evidence-based practice: a new paradigm brings new opportunities for health sciences librarians. Med Ref Serv Q. 2005 Winter;24(4):1–16.
198. Perry GJ, Roderer NK, Assar S. A current perspective on medical informatics and health sciences librarianship. J Med Libr Assoc. 2005 Apr;93(2):199–205.
199. Phillips RM, Bonsteel SH. The faculty and information specialist partnership: stimulating student interest and experiential learning. Nurse Educ. 2010 May;35(3):136–8. DOI: http://dx.doi.org/10.1097/NNE.0b013e3181d95090.
200. Phinney J, Horsman AR. Satellite stories: capturing professional experiences of academic health sciences librarians working in delocalized health sciences programs. J Med Libr Assoc. 2018 Jan;106(1):74–80. DOI: http://dx.doi.org/10.5195/jmla.2018.214.
201. Plutchak TS. Breaking the barriers of time and space: the dawning of the great age of librarians. J Med Libr Assoc. 2012 Jan;100(1):10–9. DOI: http://dx.doi.org/10.3163/1536-5050.100.1.004.
202. Rader T, Gagnon AJ. Expediting the transfer of evidence into practice: building clinical partnerships. Bull Med Libr Assoc. 2000 Jul;88(3):247–50.
203. Raimondo PG, Harris RL, Nance M, Brown ED. Health literacy and consent forms: librarians support research on human subjects. J Med Libr Assoc. 2014 Jan;102(1):5–8. DOI: http://dx.doi.org/10.3163/1536-5050.102.1.003.
204. Rankin JA, Grefsheim SF, Canto CC. The emerging informationist specialty: a systematic review of the literature. J Med Libr Assoc. 2008 Jul;96(3):194–206. DOI: http://dx.doi.org/10.3163/1536-5050.96.3.005.

205. Reinbold S. Using the ADDIE model in designing library instruction. Med Ref Serv Q. 2013 Jul;32(3):244–56.
206. Robb BG, Zipperer L. Knowledge management in hospitals: drawing from experience to define the librarian's role. J Hosp Librariansh. 2009;9(3):307–17.
207. Roper FW. The Medical Library Association's professional development program: a look back at the way ahead. J Med Libr Assoc. 2006 Jan;94(1):8–18.
208. Rossini B, Burnham J, Wright A. The librarian's role in an enrichment program for high school students interested in the health professions. Med Ref Serv Q. 2013;32(1):73–83.
209. Saimbert MK, Zhang Y, Pierce J, Moncrief ES, O'Hagan KB, Cole P. Medical librarians supporting information systems project lifecycles toward improved patient safety. medical librarians possess expertise to navigate various search resources and can investigate inquiries during IS project lifecycles. J Healthc Inf Manag. 2010 Winter;24(1):52–6.
210. Sathe NA, Jerome R, Giuse NB. Librarian-perceived barriers to the implementation of the informationist/information specialist in context role. J Med Libr Assoc. 2007 Jul;95(3):270–4. DOI: http://dx.doi.org/10.3163/1536-5050.95.3.270
211. Schardt C. Evidence-based medicine and the hospital librarian. J Hosp Librariansh. 2001;1(2):1–14.
212. Schardt C. Health information literacy meets evidence-based practice [editorial]. J Med Libr Assoc. 2011 Jan;99(1):1–2. DOI: http://dx.doi.org/10.3163/1536-5050.99.1.001.
213. Scherrer CS. Evaluating a health sciences library residency program: what have we learned? J Med Libr Assoc. 2010 Oct;98(4):300–2. DOI: http://dx.doi.org/10.3163/1536-5050.98.4.006.
214. Scherrer CS, Jacobson S. New measures for new roles: defining and measuring the current practices of health sciences librarians. J Med Libr Assoc. 2002 Apr;90(2):164–72.
215. Schnitzer AE, Rosenzweig M, Harris B. Health literacy: a survey of the issues and solutions. J Consumer Health Internet. 2011;15(2):164–79.
216. Schwing LJ, Coldsmith EE. Librarians as hidden gems in a clinical team. Med Ref Serv Q. 2005 Spring;24(1):29–39.
217. Scola-Streckenbach S. Experience-based information: the role of web-based patient networks in consumer health information services. J Consumer Health Internet. 2008;12(3):216–36.
218. Shachak A, Shuval K, Fine S. Barriers and enablers to the acceptance of bioinformatics tools: a qualitative study. J Med Libr Assoc. 2007 Oct;95(4):454–8. DOI: http://dx.doi.org/10.3163/1536-5050.95.4.454.
219. Shearer BS, Seymour A, Capitani C. Bringing the best of medical librarianship to the patient team. J Med Libr Assoc. 2002 Jan;90(1):22–31.
220. Sheffield C. e-Learning Object Portals: a new resource that offers new opportunities for librarians. Med Ref Serv Q. 2006 Winter;25(4):65–74.
221. Shipman JP. Why emerging roles for health sciences librarians? Ref Serv Rev. 2004;32(1):9–12.
222. Shipman JP, Kurtz-Rossi S, Funk CJ. The Health Information Literacy Research Project. J Med Libr Assoc. 2009 Oct;97(4):293–301. DOI: http://dx.doi.org/10.3163/1536-5050.97.4.014.
223. Shipman JP, Stoddart JM, Peay WJ, Eccles SS. Spaces that support redefined roles of academic health sciences librarians. J Med Libr Assoc. 2013 Jul;101(3):179–84. DOI: http://dx.doi.org/10.3163/1536-5050.101.3.006.
224. Shipman JP, Watstein SB. Emerging roles of health sciences librarians. Ref Serv Rev. 2004;32(1):8–92.

225. Shurtz S, Foster MJ. Developing and using a rubric for evaluating evidence-based medicine point-of-care tools. J Med Libr Assoc. 2011 Jul;99(3):247–54. DOI: http://dx.doi.org/10.3163/1536-5050.99.3.012.
226. Simpson SN, Coghill JG, Greenstein PC. The electronic resources librarian in the health sciences library: an emerging role. J Electron Resour Med Libr. 2005;2(1):27–39.
227. Sladek R, Tieman J, Fazekas BS, Abernethy AP, Currow DC. Development of a subject search filter to find information relevant to palliative care in the general medical literature. J Med Libr Assoc. 2006 Oct;94(4):394–401.
228. Smith CA. An evolution of experts: MEDLINE in the library school. J Med Libr Assoc. 2005 Jan;93(1):53–60.
229. Sokolow D. You want me to do what? medical librarians and the management of archival collections. J Hosp Librariansh. 2004;4(4):31–50.
230. Sollenberger JF, Holloway Jr RG. The evolving role and value of libraries and librarians in health care. JAMA. 2013 Sep 25;310(12):1231–2.
231. Starr S. Improving instruction in medical libraries: the research agenda [editorial]. J Med Libr Assoc. 2012 Oct;100(4):236–8. DOI: http://dx.doi.org/10.3163/1536-5050.100.4.003.
232. Starr S. The librarian in the cloud: or beware of unintended consequences [editorial]. J Med Libr Assoc. 2011 Oct;99(4):267–9. DOI: http://dx.doi.org/10.3163/1536-5050.99.4.001.
233. Steelman SC, Thomas SL. Academic health sciences librarians' contributions to institutional animal care and use committees. J Med Libr Assoc. 2014 Jul;102(3):215–9. DOI: http://dx.doi.org/10.3163/1536-5050.102.3.014.
234. Stellrecht E, Chiarella D. Targeted evolution of embedded librarian services: providing mobile reference and instruction services using iPads. Med Ref Serv Q. 2015;34(4):397–406.
235. Stephenson PL, Clever S, Coady TR, Ender D, Heyd M, Peth S. Book clubs-outreach opportunities for hospital libraries. Med Ref Serv Q. 2014;33(4):448–59.
236. Stephenson PL, Green BF, Wallace RL, Earl MF, Orick JT, Taylor MV. Community partnerships for health information training: medical librarians working with health-care professionals and consumers in Tennessee. Health Inf Libr J. 2004;21:20–6.
237. Sulimanoff I, Hernandez M, Gibson DS. The clinical medical librarian program: the Memorial Sloan-Kettering experience. J Hosp Librariansh. 2011;11(4):338–47.
238. Sullo E, Gomes AW. A profession without limits: the changing role of reference librarians. Med Ref Serv Q. 2016;35(2):145–57.
239. Tan MC, Maggio LA. Expert searcher, teacher, content manager, and patient advocate: an exploratory study of clinical librarian roles. J Med Libr Assoc. 2013 Jan;101(1):63–72. DOI: http://dx.doi.org/10.3163/1536-5050.101.1.010.
240. Tarver T. Genomics: a new challenge in consumer health information literacy. J Hosp Librariansh. 2010;10(1):23–32.
241. Tarver T, Jones DA, Adams M, Garcia A. The librarian's role in linking patients to their personal health data and contextual information. Med Ref Serv Q. 2013 Oct;32(4):459–67. DOI: http://dx.doi.org/10.1080/02763869.2013.837730.
242. Taylor MV. Impressions of an old master: hospital libraries and librarians, 1970–2014. Med Ref Serv Q. 2015;34(1):104–12.
243. Teal J, Wax D, Eldredge J, Hendrix I. Instructional objectives: a means to designing effective instructional sessions for hospital librarians. J Hosp Librariansh. 2004;4(2):47–56.

© Ma, Stahl, Knotts 2018

244. Tennant MR, Auten B, Botero CE, Butson LC, Edwards ME, Garcia-Milian R, Lyon JA, Norton HF. Changing the face of reference: adapting biomedical and health information services for the classroom, clinic, and beyond. Med Ref Serv Q. 2012 Jul;31(3):280–301. DOI: http://dx.doi.org/10.1080/02763869.2012.698172.
245. Tennant MR, Butson LC, Rezeau ME, Tucker PJ, Boyle ME, Clayton G. Customizing for clients: developing a library liaison program from need to plan. Bull Med Libr Assoc. 2001 Jan;89(1):8–20.
246. Tennant MR, Edwards M, Miyamoto MM. Use of instructional design theory and an individualized hybrid strategy for assessment in library-based instruction. J Med Libr Assoc. 2012 Oct;100(4):319–22. DOI: http://dx.doi.org/10.3163/1536-5050.100.4.018.
247. Thibodeau PL, Funk CJ. Trends in hospital librarianship and hospital library services: 1989 to 2006. J Med Libr Assoc. 2009 Oct;97(4):273–9. DOI: http://dx.doi.org/10.3163/1536-5050.97.4.011.
248. Tomlin A. Hospital librarians and the Johns Hopkins tragedy. J Hosp Librariansh. 2002;2(4):89–96.
249. Traditi LK, Le Ber JM, Beattie M, Meadows SE. From both sides now: librarians' experiences at the Rocky Mountain Evidence-Based Health Care Workshop. J Med Libr Assoc. 2004 Jan;92(1):72–7.
250. Volesko MM. It wasn't raining when Noah built the ark: disaster preparedness for hospitals and medical librarians post September 11. Internet Ref Serv Q. 2001;6(3/4):99–131.
251. Volk RM. Expert searching in consumer health: an important role for librarians in the age of the Internet and the web. J Med Libr Assoc. 2007 Apr;95(2):203–7, e66. DOI: http://dx.doi.org/10.3163/1536-5050.95.2.203.
252. Walton L, Childs C, Egeland M, Brooks MK, Zipperer L. Empowering patient safety outreach through interprofessional partnerships: educating our communities. J Hosp Librariansh. 2010;10(3):224–34.
253. Watstein SB. Emerging roles of health sciences librarians: academic library centrality. Ref Serv Rev. 2004;32(1):13–5.
254. Watstein SB. Emerging roles of health sciences librarians. part 2. Ref Serv Rev. 2005;33(1):7–53.
255. Weaver D. Enhancing resident morning report with "daily learning packages." Med Ref Serv Q. 2011;30(4):402–10.
256. Weise FO, McMullen TD. Study to assess the compensation and skills of medical library professionals relative to information technology professionals. Bull Med Libr Assoc. 2001 Jul;89(3):249–62.
257. Welton NJ. The University of Washington electronic medical record experience. J Med Libr Assoc. 2010 Jul;98(3):217–9. DOI: http://dx.doi.org/10.3163/1536-5050.98.3.008.
258. Wessel CB, Tannery NH, Epstein BA. Evaluation of a self-paced learning module to teach responsible literature searching for research. J Med Libr Assoc. 2010 Jan;98(1):82–5. DOI: http://dx.doi.org/10.3163/1536-5050.98.1.020.
259. Whitmore SC, Grefsheim SF, Rankin JA. Informationist programme in support of biomedical research: a programme description and preliminary findings of an evaluation. Health Inf Libr J. 2008;25(2):135–41.
260. Williams J, McCrillis A, McGowan R, Nicholson J, Surkis A, Thompson H, Vieira D. Leveraging technology and staffing in developing a new liaison program. Med Ref Serv Q. 2014;33(2):157–66.
261. Williams L, Zipperer L. Improving access to information: librarians and nurses team up for patient safety. Nurs Econ. 2003 Jul–Aug;21(4):199–201.
262. Wolf DG, Chastain-Warheit CC, Easterby-Gannett S, Chayes MC, Long BA. Hospital librarianship in the United States: at the crossroads. J Med Libr Assoc. 2002 Jan;90(1):38–48.

JMLA Journal of the Medical Library Association

SC
Supplemental content to
J Med Libr Assoc. Oct;106(4):dx.doi.org/10.5195/jmla.2018.354
www.jmla.mlanet.org
© Ma, Stahl, Knotts 2018

263. Wu L, Li P. What do they want? a content analysis of Medical Library Association reference job announcements, 2000–2005. J Med Libr Assoc. 2008 Oct;96(4):378–81. DOI: http://dx.doi.org/10.3163/1536-5050.96.4.015.
264. Wu L, Mi M. Sustaining librarian vitality: embedded librarianship model for health sciences libraries. Med Ref Serv Q. 2013;32(3):257–65.
265. Wyles DF. Connecting families and community resources: the librarian's role in pediatric advocacy. J Hosp Librariansh. 2009;9(1):1–7.
266. Zeblisky K, Birr RA, Sjursen G. Effecting change in an evidence-based medicine curriculum: librarians' role in a pediatric residency program. Med Ref Serv Q. 2015;34(3):370–81.
267. Zeblisky KA, Birr RA, Aguiñaga AL, Drachman D, Mathieson K. Rethinking your involvement: a survey on hospital library committee participation. J Hosp Librariansh. 2013;13(1):47–58.
268. Zipperer L, Gillaspy M, Goeltz R. Facilitating patient centeredness through information work: seeing librarians as guests in the lives of patients. J Hosp Librariansh. 2005;5(3):1–15.

Note: Articles of the nine categories, grouped and saved in an Endnote file, are available by request.
